# A Meta‐Analysis to Unveil the Diagnostic Gaps in Anderson–Fabry Disease in Women

**DOI:** 10.1002/jimd.70153

**Published:** 2026-02-05

**Authors:** L. Lenzini, G. Pintus, G. Gugelmo, A. P. Burlina, G. P. Fadini, A. B. Burlina, N. Vitturi

**Affiliations:** ^1^ Department of Medicine University of Padova Padova Italy; ^2^ Nephrology and Dialysis, Department of Medical Sciences Tor Vergata University Hospital Roma Italy; ^3^ Division of Metabolic Diseases, Department of Medicine Padova University Hospital, University of Padova Padova Italy; ^4^ Neurology Unit, St. Bassiano Hospital Bassano del Grappa Italy; ^5^ Division of Inherited Metabolic Diseases, Department of Women's and Children's Health University of Padova Padova Italy

**Keywords:** Anderson–Fabry disease, diagnosis, gender medicine, genetic screening, high risk population, inherited metabolic disease, meta‐analysis

## Abstract

Anderson–Fabry disease (AFD) is an X‐linked lysosomal storage disorder caused by mutations in the GLA gene, leading to deficient α‐galactosidase A activity. Although historically considered a male disease, it is now recognized that heterozygous women can present with a wide range of symptoms. However, diagnosis in women remains challenging, as enzymatic activity may be normal. A meta‐analysis of 67 studies was conducted to evaluate the prevalence of AFD in female populations referred for cardiac, renal, or cerebrovascular events of unknown etiology, focusing on current diagnostic methodologies. Out of 28 878 high‐risk women screened, 114 were diagnosed with AFD, yielding a pooled prevalence of 0.007 (95% CI 0.005–0.009). When considering the prevalence in the three main groups of indication for AFD testing (patients referred for cardiac, renal or cerebrovascular events of unknown etiology), the highest prevalence was found after a stroke (23 studies: 0.014, 95% CI 0.011–0.019), followed by cardiac event (19 studies: 0.010, 95% CI 0.007–0.015) and renal event (25 studies: 0.004, 95% CI 0.003–0.006). Genetic testing was significantly more effective in identifying cases (prevalence of AFD from 34 studies: 0.012, 95% CI 0.010–0.015) compared to enzymatic protocols (prevalence of AFD from 33 studies: 0.003, 95% CI 0.002–0.005). However, the reliance on enzymatic testing in some regions is still preferred. These findings underscore the limitations of relying solely on enzymatic assays in women and highlight the critical role of genetic testing in achieving accurate diagnosis. Early identification allows for timely treatment and enables family cascade screening, preventing further missed diagnoses.

## 1 Introduction

Anderson–Fabry disease (AFD) is an X‐linked lysosomal storage disorder caused by pathogenic variants in the *GLA* gene, leading to deficient activity of the enzyme α‐galactosidase A. This enzymatic deficiency results in the progressive accumulation of globotriaosylceramide (Gb3) and related glycosphingolipids in multiple organ systems, contributing to significant morbidity and mortality [1]. While classically considered a disease affecting hemizygous males, it is now well recognized that heterozygous females can also exhibit a broad spectrum of clinical manifestations due to X‐chromosome inactivation and variable enzyme activity levels [2, 3]. Early and accurate diagnosis in this group of patients is critical for timely initiation of enzyme replacement therapy (ERT) or chaperone therapy, which can help mitigate disease progression [4].

The availability of therapy has increased awareness and implemented population‐based and high‐risk screening studies to identify undiagnosed cases of AFD, particularly among individuals presenting with suggestive clinical features such as unexplained left ventricular hypertrophy, chronic kidney disease of unknown origin, or cryptogenic stroke. Although females are generally less severely affected, it is now recognized that they cannot be considered simply carriers as they display the full clinical picture of the disease [5].

However, the diagnostic approach to AFD in women remains challenging due to variable enzymatic activity, necessitating the use of genetic testing to confirm diagnosis.

AFD can be diagnosed by quantifying α‐galactosidase A activity in blood, using dried blood spot (DBS) tests, or in leukocytes, or by identifying a causative variant of the a‐Gal A gene. More recently, Lyso‐Gb3 quantification has also been used as a biomarker for the diagnosis of AFD, aiding in the identification of affected individuals, particularly in cases with atypical symptoms or late‐onset variants [6].

In male hemizygotes a reduced a‐Gal A activity is always present, while 40%–70% of female carriers have a normal a‐Gal A activity [7, 8, 9].

Thus, genetic testing should be performed in women by sequencing the entire *GLA* gene, due to the high number of variants of the *GLA* gene found in AFD.

However, diagnostic approaches to AFD in women vary significantly across the globe. This is particularly evident in screening studies aimed at uncovering the underlying causes of unexplained cerebrovascular, cardiac, and renal disorders. These differing strategies reflect the challenges associated with the often subtle and atypical clinical presentation of AFD in female patients, which can lead to underdiagnosis or delayed recognition of the disease.

The primary objectives of this meta‐analysis are twofold: (1) to evaluate the difference in prevalence of AFD diagnoses among women in high‐risk populations when using DNA testing compared to enzymatical and biochemical testing and (2) to assess what the most commonly applied testing protocols in the literature are, with a classification based on geographical distribution and temporal trends.

By analyzing available screening data in high‐risk individuals, this study aims to provide a comprehensive assessment of current diagnostic methodologies and their implications for identifying AFD in women. Understanding these differences is crucial for optimizing screening strategies and improving diagnostic accuracy in this at‐risk population.

## 2 Methods

### 2.1 Protocol and Registration

This systematic review and metanalysis was developed according to the preferred reporting items for systematic reviews and meta‐analyses (PRISMA) guidelines [10]. The protocol was registered in the International Prospective Register of Systematic Reviews (PROSPERO), registration number [CRD42025643472] (https://www.crd.york.ac.uk/prospero/).

### 2.2 Search Strategy and Eligibility Criteria

Two reviewers conducted a literature search of PubMed in January 2025. The following terms were used: (prevalence OR frequency OR screening) AND (fabry disease OR fabry‐disease OR anderson fabry disease OR anderson‐fabry disease OR fabry) NOT (case report OR review OR meta‐analysis) AND (women OR females) AND (dialysis OR hemodialysis OR renal screening OR chronic kidney disease OR end stage renal disease OR kidney replacement therapy OR kidney transplantation OR stroke cardiac OR heart OR left ventricular hypertrophy OR hypertrophic cardiomyopathy OR cardiac magnetic resonance OR echocardiography OR arrhythmias OR pacemaker OR defibrillator OR atrial fibrillation OR myocardial infarction OR ventricular arrhythmias OR cardiac arrest OR ventricular). The search was restricted to studies in English.

Inclusion criteria were: (1) studies designed as observational cohort study or cross‐sectional study and (2) prevalence data reported as number of confirmed cases over sample size, on an individual and sex basis; Exclusion criteria were: (1) the study population should not have been newborns; (2) the study should not have preselected cohorts; (3) the study should have reported the description of the diagnostic test; and (4) the *GLA* variant should have been reported for each case.

Additional screening studies were found by crosschecking references. Two authors (L.L. and G.G.) independently screened the studies, obtained the full reports of potentially relevant studies, and reviewed each paper using predefined eligibility criteria. Any discrepancy regarding the eligibility of a study was discussed with a third author (N.V.).

To classify the reported *GLA* variants we applied the guidelines from the American College of Medical Genetics and Genomics (ACMG) for the assignment of pathogenicity of genetic variants and to exclude studies reporting diagnosis with neutral or benign *GLA* variants.

We excluded case reports, reviews, and meta‐analysis. All duplicate papers were removed.

### 2.3 Data Collection and Quality Assessment

The following data were collected for each study: first author, year of publication, female and male sample size, number of confirmed female and male cases, screening indication, screening and confirmatory test, gender, and *GLA* variants identified (Table S1).

The risk of bias of the included studies was assessed using a quality assessment tool appropriate for prevalence studies (10 items that addressed four domains of bias including target population, random selection of participants, and quality of data collection) [11]. Two authors (L.L. and G.G.) independently assessed the selected studies for the risk of bias. Any discrepancies were solved through discussion with a third author (N.V.).

### 2.4 Meta‐Analysis of Included Studies

For each study, prevalence was calculated in females. To account for zero‐event cells, we used the continuity correction for handling zero‐event data. Random‐effects meta‐analysis was used to pool the data, using the DerSimonian and Laird method to perform the variance estimation. *Q*‐test for heterogeneity, *I* ^2^ statistic, *T* ^2^, and *T* were calculated to assess quality of the studies. Publication bias was checked by funnel plot analysis. All meta‐analytical calculations were performed using Comprehensive Meta‐analysis 4 software [12].

## 3 Results

### 3.1 Meta‐Analysis Results

**FIGURE 1:**
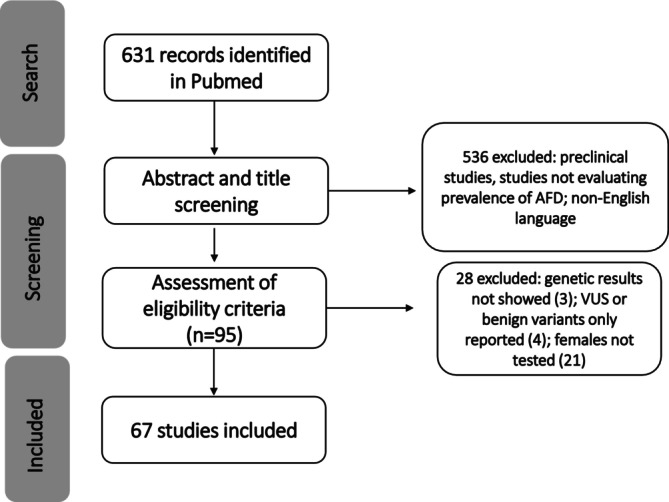
Flow‐chart of the systematic review developed according to the Preferred Reporting Items for Systematic reviews and Meta‐Analyses (PRISMA) guidelines [10].

Applying the search terms, 631 studies were initially considered and screened (Figure 1). After a first selection based on title and abstract, 95 were further screened looking at the full text. Among them, 28 were excluded because the genetic results were not shown (3) or only VUS or benign variants were reported (4) or females were not tested (21) (Table S2). Sixty seven studies (Table 1) were finally selected according to the inclusion criteria.

**TABLE 1:** Studies included in the meta‐analysis.

Study name	Positive case (*n*)	Sample size (*n*)	Year	Referral cause	Country	Continent	Screening test
Afanasiev, 2020 [13]	1	39	2020	STROKE	Israel	EU	Genetic test
Aladağ, 2023 [14]	0	69	2023	CARDIAC	Turkey	EU	Genetic test
Arad, 2005 [15]	0	8	2005	CARDIAC	USA	AMERICA	Genetic test
Baptista, 2010 [16]	5	192	2010	STROKE	Portugal	EU	Genetic test
Barman, 2019 [17]	0	27	2019	CARDIAC	Turkey	EU	Genetic test
Batta, 2024 [18]	1	40	2024	RENAL	India	ASIA	Enzymatic test
Bekri 2005 [19]	0	47	2005	CARDIAC	France	EU	Enzymatic test
Braga Silva, 2024 [20]	7	1874	2024	RENAL	Brasil	AMERICA	Enzymatic test
Brouns, 2007 [21]	0	39	2007	STROKE	Belgium	EU	Enzymatic test
Brouns, 2010 [22]	8	448	2010	STROKE	Belgium	EU	Genetic test
De Schoenmakere, 2008 [23]	0	395	2008	RENAL	Belgium	EU	Enzymatic test
Dubuc, 2013 [24]	0	45	2013	STROKE	Canada	AMERICA	Genetic test
Elliott, 2011 [25]	4	501	2011	CARDIAC	UK	EU	Genetic test
Erdogmus, 2020 [26]	2	120	2020	RENAL	Turkey	EU	Genetic test
Fancellu, 2015 [27]	1	105	2015	STROKE	Italy	EU	Genetic test
Fujii, 2009 [28]	2	399	2009	RENAL	Japan	ASIA	Enzymatic test
Gaspar, 2010 [29]	3	368	2010	RENAL	Spain	EU	Enzymatic test
Gündoğdu, 2017 [30]	0	30	2017	STROKE	Turkey	EU	Enzymatic test
Hagège, 2011 [31]	0	114	2011	CARDIAC	France	EU	Enzymatic test
Härtl, 2023 [32]	0	60	2023	STROKE	Germany	EU	Genetic test
Havndrup, 2010 [33]	1	34	2010	CARDIAC	Denmark	EU	Genetic test
Imasawa, 2023 [34]	0	888	2023	RENAL	Japan	ASIA	Enzymatic test
Jahan, 2020 [35]	0	201	2020	RENAL	Australia	OCEANIA	Enzymatic test
Kilarski, 2015 [36]	0	288	2015	STROKE	UK	EU	Genetic test
Kinoshita, 2018 [37]	0	120	2018	STROKE	Japan	ASIA	Enzymatic test
Kljajic, 2024 [38]	0	334	2024	RENAL	Croatia	EU	Genetic test
Kotanko, 2004 [39]	0	964	2004	RENAL	Austria	EU	Enzymatic test
Lanthier, 2017 [40]	2	179	2017	STROKE	Canada	AMERICA	Genetic test
Lau, 2025 [41]	0	37	2025	CARDIAC	Germany	EU	Genetic test
Lee, 2019 [42]	0	240	2019	STROKE	Taiwan	ASIA	Genetic test
Leung, 2024 [43]	2	113	2024	CARDIAC	China	ASIA	Enzymatic test
Lin, 2024 [44]	1	317	2024	CARDIAC	China	ASIA	Enzymatic test
Lin, 2025 [45]	0	318	2025	STROKE	China	ASIA	Enzymatic test
Lv, 2009 [46]	0	786	2009	RENAL	China	ASIA	Enzymatic test
Malavera, 2020 [47]	1	23	2020	STROKE	Australia	OCEANIA	Genetic test
Mallett, 2022 [48]	1	1259	2022	RENAL	Australia	OCEANIA	Enzymatic test
Maron, 2018 [49]	1	172	2018	CARDIAC	USA	AMERICA	Genetic test
Marquardt, 2012 [50]	4	544	2012	STROKE	UK	EU	Genetic test
Merta, 2006 [51]	1	1849	2006	RENAL	Czech Republic	EU	Enzymatic test
Moiseev, 2019 [52]	1	2021	2019	RENAL	Russia	ASIA	Enzymatic test
Monserrat, 2007 [53]	0	180	2007	CARDIAC	Spain	EU	Enzymatic test
Nagamatsu, 2017 [54]	1	225	2017	STROKE	Japan	ASIA	Enzymatic test
Nishino, 2012 [55]	2	376	2012	RENAL	Japan	ASIA	Enzymatic test
Okur, 2013 [56]	0	521	2013	RENAL	Turkey	EU	Enzymatic test
Ozpelit, 2023 [57]	1	107	2023	CARDIAC	Turkey	EU	Genetic test
Poli, 2017 [58]	2	158	2017	STROKE	Italy	EU	Genetic test
Reisin, 2018 [59]	3	143	2018	STROKE	Argentina	AMERICA	Genetic test
Reynolds, 2020 [60]	1	579	2020	RENAL	UK	EU	Enzymatic test
Romani, 2024 [61]	7	634	2024	STROKE	Italy	EU	Genetic test
Romani, 2015 [62]	2	42	2015	STROKE	Italy	EU	Genetic test
Sadasivan, 2020 [63]	0	99	2020	CARDIAC	Canada	AMERICA	Enzymatic test
Saito, 2016 [64]	5	3139	2016	RENAL	Japan	ASIA	Enzymatic test
Sarikaya, 2012 [65]	0	48	2012	STROKE	Switzerland	EU	Genetic test
Savostyanov, 2022 [66]	3	431	2022	CARDIAC	Russia	ASIA	Genetic test
Sens, 2024 [67]	1	1094	2024	RENAL	France	EU	Enzymatic test
Song, 2016 [68]	0	64	2016	STROKE	China	ASIA	Genetic test
Terryn, 2008 [69]	2	742	2008	RENAL	Belgium	EU	Enzymatic test
Terryn, 2013 [70]	3	178	2013	CARDIAC	Belgium	EU	Genetic test
Tomek, 2021 [71]	7	450	2021	STROKE	Czeck Republic	EU	Genetic test
Tran Vu, 2019 [72]	0	38	2019	CARDIAC	Vietnam	ASIA	Genetic test
Turkmen, 2016 [73]	0	146	2016	RENAL	Turkey	EU	Enzymatic test
Yalin, 2019 [74]	21	2233	2019	RENAL	Turkey	EU	Genetic test
Yamashita S, 2019 [75]	0	62	2019	CARDIAC	Japan	ASIA	Enzymatic test
Yeniçerioğlu 2017 [76]	0	656	2017	RENAL	Turkey	EU	Enzymatic test
Yilmaz, 2017 [77]	2	648	2017	RENAL	Turkey	EU	Genetic test
Zemanek, 2022 [78]	2	199	2022	CARDIAC	Czech Republic	EU	Enzymatic test
Zizzo, 2018 [79]	0	79	2018	RENAL	Italy	EU	Genetic test

Globally, heterogeneity was analyzed among the studies by comparing the *Q*‐value with the degrees of freedom, resulting in 116 142 with 66 degrees of freedom and *p* < 0.001, which likely indicates the heterogeneity due to the use of different testing methods. The *I* ^2^ statistic indicated that 43% of the variance in observed effects reflects variance in true effects rather than sampling error. *T* ^2^ was 0.414 and *T* was 0.643.

However, when heterogeneity was assessed grouping studies according to genetic or enzymatic testing method, it decreased in both groups: *Q*‐value: 28.865, with dF: 33, *p* = 0.673; *I* ^2^: 0.000; *T* ^2^: 0.000, and *T*: 0.000 (genetic tests); *Q*‐value: 40.053, with dF: 32, *p* = 0.155; *I* ^2^: 20.106; *T* ^2^: 0.209, and *T*: 0.457 (enzymatic tests).

Publication bias was assessed globally (Figure S1A) or according to testing methods (Figure S1B,C) by funnel plot analysis. The risk of bias of included studies is reported in Table S3. Overall, 70% of studies were rated as low risk; none was at high risk. Enzymatic assays outperformed genetic assays in methodological quality. Studies in cardiac and renal categories demonstrated higher proportions of low‐risk methodology compared to studies in stroke cohorts.

### 3.2 Prevalence of AFD in High‐Risk Female Populations

**FIGURE 2:**
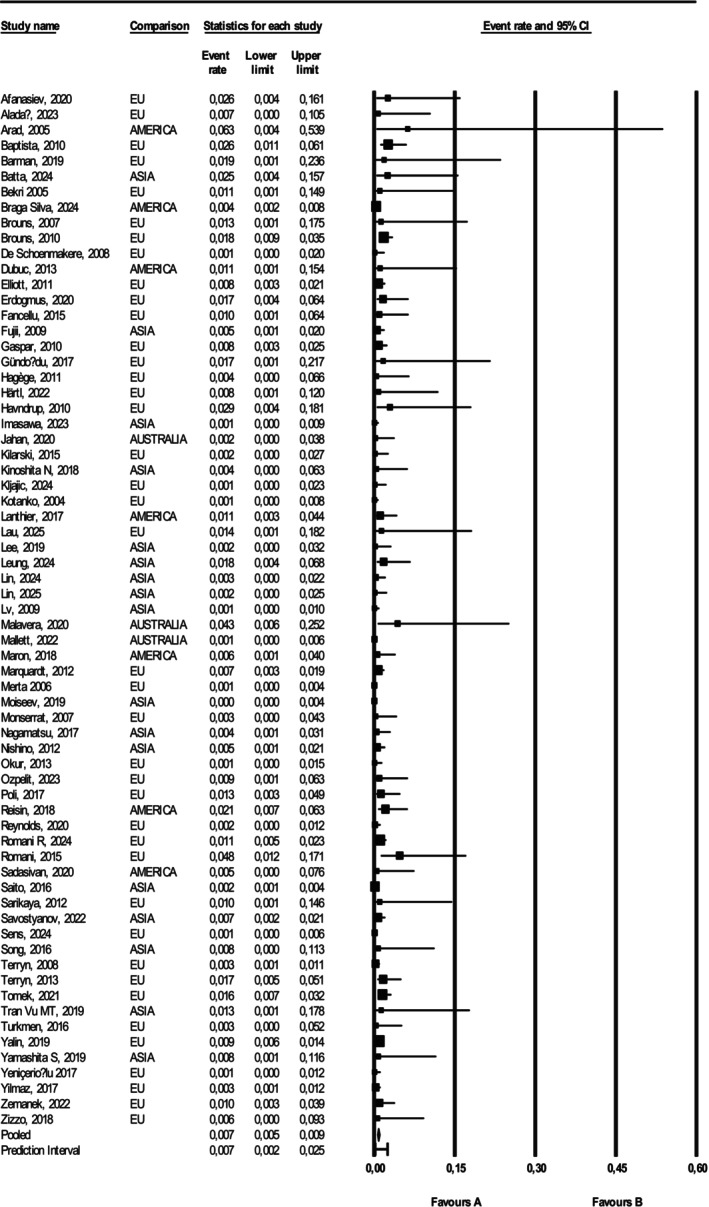
Forest plot of the studies included in the meta‐analysis. To pool the data, random‐effects meta‐analysis was used, using the DerSimonian and Laird method to perform the variance estimation with Comprehensive Meta‐analysis 4 software [12].

Globally, 28 878 women were tested among patients referred to screening after a cardiac or renal or cerebrovascular event of unknown etiology (Figure 2). One‐hundred and fourteen female subjects were diagnosed with AFD, with a pooled prevalence of 0.007 (95% CI 0.005–0.009) of female subjects.

When considering the prevalence in the three main groups of indication for AFD testing (patients referred for cardiac, renal or cerebrovascular events of unknown etiology), it was significantly different among groups (*p* < 0.001) (Table 2), with the highest prevalence found after a stroke (44 positive cases on 4434 screened subjects in 23 studies: 0.014, 95% CI 0.011–0.019), followed by cardiac event (18 positive cases on 2733 screened subjects in 19 studies: 0.010, 95% CI 0.007–0.015) and renal event (52 positive cases on 21 711 screened subjects in 25 studies: 0.004, 95% CI 0.003–0.006).

**TABLE 2:** Pooled prevalence of AFD diagnosis in high‐risk populations.

	Prevalence	Positive case (*n*)	Sample size (*n*)	Studies (*n*)
All populations	0.007 (0.005–0.009)	114	28 878	67
Screened for cardiac events	0.010 (0.007–0.015)	18	2733	19
Screened for renal diseases	0.003 (0.002–0.004)	52	21 711	25
Screened for stroke	0.014 (0.011–0.019)	44	4434	23

The pooled prevalence of AFD diagnosis was 0.004 (18 positive cases on 9577 screened subjects in 17 studies, 95% CI 0.003–0.006) in Asia, 0.010 (81 positive cases on 15 298 screened subjects in 40 studies, 95% CI 0.008–0.012) in Europe, 0.007 (13 positive cases on 2520 screened subjects in 7 studies, 95% CI 0.004–0.012) in America, and 0.005 (two positive cases on 1483 screened subjects in three studies, 95% CI 0.001–0.017) in Oceania.

### 3.3 Prevalence of AFD in High‐Risk Female Populations According to Diagnostic Protocol

**FIGURE 3:**
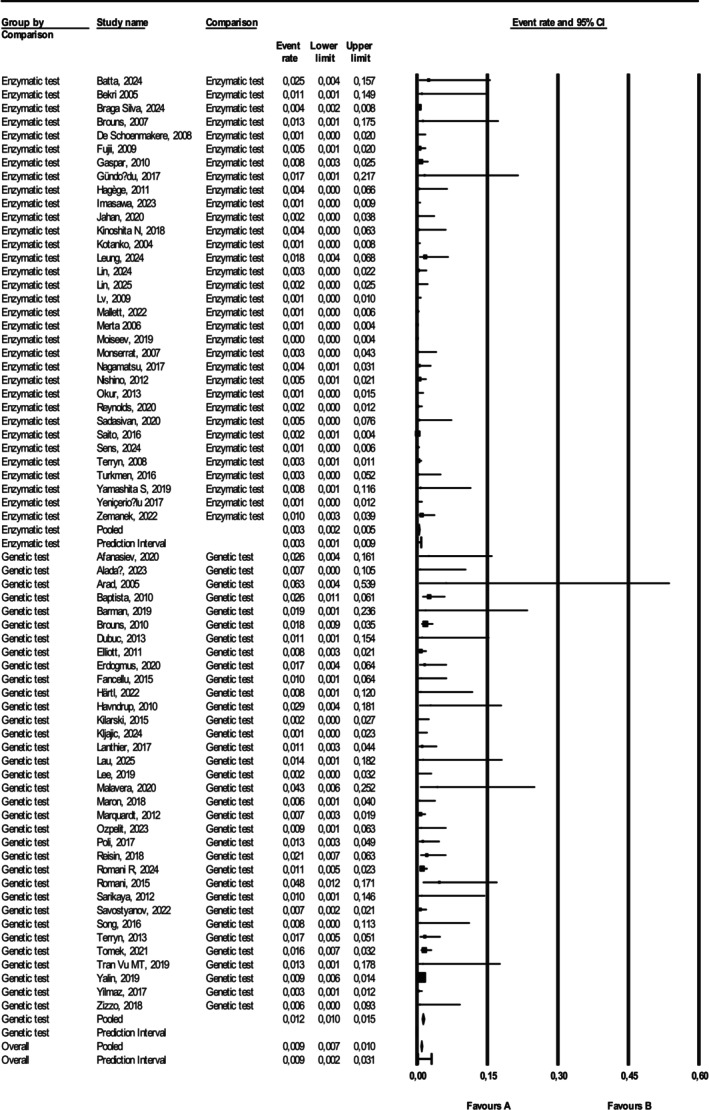
Forest plot of the studies classified according to testing method (genetic or enzymatic). To pool the data, random‐effects meta‐analysis was used, using the DerSimonian and Laird method to perform the variance estimation with Comprehensive Meta‐analysis 4 software [12].

In the 34 studies using genetic test to search for AFD, the pooled prevalence was 0.012 (81 positive cases on 8718 screened subjects, 95% CI 0.010–0.015), with 21 studies reporting at least 1 diagnosed case; while in the other 33 studies, where different enzymatic and biochemical protocols were followed, the prevalence was significantly lower = 0.003 (33 positive cases on 20 160 screened subjects, 95% CI 0.002–0.005, *p* < 0.001), with 16 of the studies reporting at least one diagnosed case (Figure 3).

Considering the reported diagnostic methodologies of the studies that diagnosed at least a case of female AFD using an enzymatic approach (Table 3), the majority of studies followed a protocol entailing only the measurement of AGAL activity on DBS (9 out of 16), with the genetic test as a second test in preselected patients. Lyso‐Gb3 quantification is more often used in combination with AGAL activity than as a second test (6 out of 16).

**TABLE 3:** Characteristics of the protocols of included studies using an enzymatic and biochemical test and reporting at least one positive AFD female case.

Study name	Sample size (*n*)	First screening test	Material	Cut‐off for positivity	Positive at first test (*n*)	Second screening test	Positive at second test	Third screening test	Positive at confirmatory test (*n*)
Zemanek, 2022 [78]	199	AGAL activity and Lyso‐Gb3	Blood on DBS	< 1.2 μmol/h/L; > 3.5 ng/mL	6	Genetic test	2	Genetic test	
Merta, 2006 [51]	1849	AGAL activity	Blood on DBS and leukocytes	< 1.5 nmol/h/mL	3	Genetic test	1		
Terryn, 2008 [69]	742	AGAL activity	Blood on DBS	< 1.6 mmol/L/h	NA	Genetic test	2		
Fujii, 2009 [28]	399	AGAL activity	Blood on DBS	< 20 Agal U	NA	AGAL activity	21	Genetic test	2
Gaspar, 2010 [29]	368	AGAL activity	Blood on DBS	< 0.5 nmol/h/spot	125	Genetic test	3		
Moiseev, 2021 [52]	2021	AGAL activity	Blood on DBS	< 1.89 μmol/L/h	NA	Genetic test	1		
Braga Silva, 2024 [20]	1874	AGAL activity and Lyso‐Gb3	Blood on DBS	< 1.68 μmol/L/h; > 2 ng/mL	132	Genetic test	7		
Reynolds, 2020 [60]	579	AGAL activity and Lyso‐Gb3	Blood on DBS	< 1.2 μmol/L/h; > 0–3.5 ng/mL	17	Genetic test	1		
Mallett, 2022 [48]	1259	AGAL activity	Blood on DBS	NA	NA	Lyso‐Gb3 Genetic test	1		
Nagamatsu^a^, 2017 [54]	225	AGAL activity	Blood on DBS	< 10 AgalU	2	Genetic test	1		
Batta, 2024 [18]	40	AGAL activity	Blood on DBS	< 3 nmol/h/dL	6	Genetic test	1		
Lin, 2024 [44]	317	AGAL activity and Lyso‐Gb3	Blood on DBS	< 2.2 μmol/h/mL; > 1.11 ng/mL	16	Genetic test	1		
Nishino, 2012 [55]	376	AGAL activity	Blood on DBS	< 20 Agal U	31	Genetic test	2		
Leung, 2024 [43]	113	AGAL activity and Lyso‐Gb3	Blood on DBS	< 1.5 μM/h; > 0.8 ng/mL	NA	Genetic test	2		
Saito^b^, 2016 [64]	3139	AGAL protein	Plasma	< 13 ng/mL	284	AGAL activity on leukocytes	101	Genetic test	5
Sens, 2024 [67]	1200	AGAL activity and Lyso‐Gb3	Blood on DBS	< 1.2 μmol/L/h; > 3.5 ng/mL	39	Genetic test	1		

^a^ Data from the screening for GLA c.196G4C (p.E66Q) variant (rs104894833) were not considered.

^b^ Genetic test was performed on 10 of the 101 subjects positive to the second test.

**FIGURE 4:**
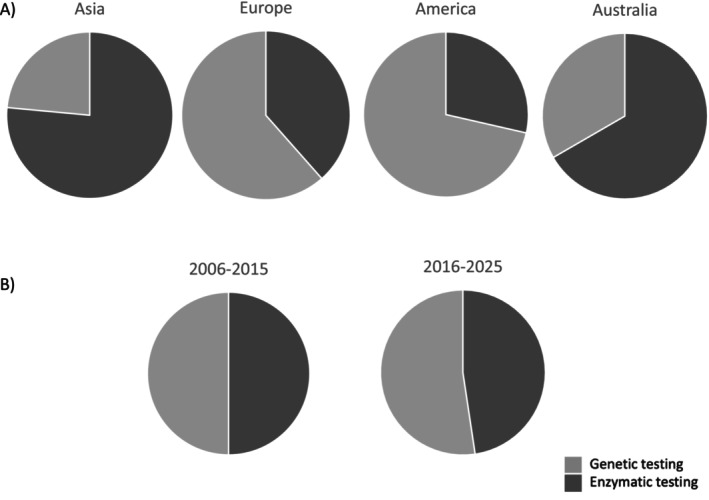
Percentage of genetic (light grey) and enzymatic (dark grey) tests performed according to continent (panel A) and years (panel B).

In Asia, the enzymatic testing was the most frequently used (76%, 13 of 17 studies), while in Europe and America it was applied in around 40% (16 of 40) and 29% (two of seven), respectively, of the studies and in two of the three studies performed in Oceania (67%) (Figure 4A).

The percentage of studies applying a diagnostic work‐up based on genetics was similar considering the temporal ranges both from 2006 to 2015 (12 studies of 25, 48%) and from 2016 until now (52%, 22/42) (Figure 4B).

## 4 Discussion

This study highlights the persistent challenges in diagnosing Anderson–Fabry Disease (AFD) in women, despite the availability of advanced diagnostic tools. Data from 28 878 female subjects referred to screening because of a cardiac, renal, or cerebrovascular event of unknown etiology emphasize that a significant number of women in high‐risk populations remain undiagnosed with a potentially treatable disease. Genetic testing emerges as the gold standard, but the global variation in diagnostic methodologies points to disparities in clinical practice. While genetic testing has proven essential for accurate diagnosis, particularly in women with variable enzymatic activity, the data collected indicate that diagnostic methodologies based solely on enzymatic and biochemical tests have led to significant underdiagnosis in this population.

In this context, it is important to note that our meta‐analysis did not include studies on neonatal screening for AFD. Although such screenings are now performed in many parts of the world [80, 81] and represent a crucial tool for the early identification of the disease in asymptomatic individuals, our aim was to provide real world data of the underdiagnosis of AFD in adult female patients with severe clinical phenotypes (i.e., overt target‐organ damage). This focus allows us to analyze in detail the diagnostic and clinical challenges of a more symptomatic and complex population, which has an immediate need for treatment and faces a high risk of multiorgan complications. A methodological limitation of the present meta‐analysis is the absence of stratified analyses according to AFD variant type or phenotype (classic versus late‐onset). It is well established that many screening cohorts are enriched for late‐onset GLA variants, such as p.N215S or IVS4, which differ substantially from classic variants in terms of clinical expression, organ involvement, and penetrance, particularly in women. Because the available studies reported variant‐ and phenotype‐specific data in a heterogeneous and often incomplete manner, a reliable stratified or descriptive synthesis could not be consistently performed without introducing additional bias.

As a consequence, the pooled prevalence estimates presented here reflect a mixture of classical and late‐onset Fabry disease variants and phenotypes. This heterogeneity may influence both the absolute prevalence values and their clinical interpretability, especially in female populations where penetrance and expressivity are highly variable. Therefore, the generalizability of these findings to specific AFD subtypes or clinical settings should be interpreted with caution. Future studies with standardized variant‐level reporting and clearer phenotypic characterization will be essential to enable stratified prevalence analyses and to better delineate the contribution of different AFD subtypes to screening outcomes.

Differently from other meta‐analyses regarding the screening in high risk populations [82, 83, 84], our study was focused only on the prevalence of AFD in women in three different high‐risk populations and considered the rate of positive diagnosis according to the applied testing method. Therefore, a direct comparison with data previously published is not straightforward.

However, this approach allowed us to highlight that, despite the cost of genetic testing having significantly decreased over the years and the availability of user‐friendly platforms continuously updated to help clinicians in the evaluation of the effects of *GLA* variants [85], about three quarters of AFD diagnoses are missed using enzymatic and biochemical tests in female subjects with a cardiovascular, renal, or cerebrovascular event of unknown etiology.

However, in this context, it should be considered that, while our prevalence estimates were intentionally restricted to variants classified as pathogenic or likely pathogenic according to current ACMG/ClinVar criteria, we acknowledge that this classification framework largely reflects a lysosomal storage–centric view of AFD pathophysiology. Recent experimental and clinical studies have clearly demonstrated that several GLA variants initially classified as benign or of uncertain significance can exert disease‐relevant effects through nonclassical mechanisms, including endoplasmic reticulum (ER) stress, protein misfolding, and proteostasis imbalance, even in the absence of overt globotriaosylceramide accumulation. These findings underscore that AFD should be viewed as a spectrum of molecular dysfunctions rather than a single storage‐driven entity, and that variant pathogenicity may evolve as mechanistic understanding advances. Variants affecting ER homeostasis represent a paradigmatic example of how AFD genetics is in continuous evolution [86, 87]. Our decision to focus the primary analysis on pathogenic/likely pathogenic variants was driven by the need for methodological consistency across cohorts; however, we recognize that this approach may underestimate the true genetic and clinical burden of AFD, especially in light of emerging ER stress‐mediated pathogenic mechanisms. These considerations highlight the importance of transparent variant‐level reporting and of interpreting prevalence estimates as dynamic constructs that will necessarily require revision as functional reclassification of GLA variants continues.

Nevertheless, the findings of this study highlight a noteworthy trend: instead of declining, the use of less expensive enzymatic assays—particularly in clinical centers across Asia—is maintained over time. This unexpected pattern suggests a persistent reliance on cost‐effective diagnostic methods in certain geographical regions, possibly reflecting local resource constraints or diagnostic preferences.

However, while initially appealing, this approach may result in missed diagnoses in women, as up to 40% of female carriers exhibit normal a‐Gal A activity [7, 8, 9]. This trade‐off between cost reduction and diagnostic accuracy remains a critical issue, particularly when addressing rare variants requiring full *GLA* gene sequencing.

The financial costs associated with undiagnosed AFD in women encompass both direct medical expenses and broader socioeconomic burdens. Without a timely diagnosis, affected women often undergo a prolonged diagnostic odyssey involving repeated specialist consultations, unnecessary procedures, and misdirected treatments. Early identification enables timely initiation of ERT or chaperone therapy, which can prevent progression and improve outcomes [4]. Moreover, the delay in initiating appropriate therapy can result in irreversible organ damage, ultimately requiring more intensive and costly interventions. This not only leads to escalating healthcare expenditures but also contributes to lost productivity, reduced quality of life, and increased dependence on long‐term healthcare services. Thus, the lack of early recognition of AFD in women imposes a significant and avoidable economic strain on both patients and healthcare systems.

The study's findings advocate for more robust screening protocols, particularly in high‐risk populations, to minimize the economic and clinical burden associated with missed diagnoses.

AFD exemplifies the importance of gender medicine in rare diseases [88]. While males typically present with more severe symptoms due to hemizygosity, women display a heterogeneous clinical spectrum that often leads to under‐diagnosis or misdiagnosis. This study further underscores the need for gender‐specific diagnostic algorithms, incorporating genetic testing as a first‐line tool for women. Moreover, gender‐specific manifestations, such as increased stroke events in females, should guide targeted screening efforts in high‐risk cohorts.

Failing to diagnose AFD in a woman has implications that extend far beyond the individual. Women often act as the index case for identifying affected family members through pedigree screening. Missing a diagnosis in a woman not only delays appropriate management for her but also prevents the timely identification of at‐risk relatives, including those who might benefit from prenatal or preconception genetic counseling. Cascade screening, facilitated by an accurate diagnosis, is essential for uncovering affected family members and enabling early interventions. This highlights the need for universal protocols prioritizing genetic testing in women with suggestive clinical features, ensuring that family screening and reproductive planning are not overlooked.

## Author Contributions

**L. Lenzini** and **N. Vitturi:** conceptualization. **L. Lenzini**, **G. Pintus**, and **G. Gugelmo:** data collection. **L. Lenzini** and **G. Pintus:** methodology. **L. Lenzini:** formal analysis. **L. Lenzini**, **G. Pintus**, and **G. Gugelmo:** writing – original draft. **A. B. Burlina**, **A. P. Burlina**, **G. P. Fadini**, and **N. Vitturi:** writing – review and editing. **L. Lenzini:** guarantor for the article.

## Funding

The authors have nothing to report.

## Disclosure

The authors have nothing to report.

## Ethics Statement

The authors have nothing to report.

## Conflicts of Interest

The authors declare no conflicts of interest.

## Supporting information


**Figure S1:** Funnel plot analysis of all (A) studies and of studies classified according to the enzymatic (B) and genetic (C) screening protocol. All meta‐analytical calculations were performed using Comprehensive Meta‐analysis 4 software.
**Table S1:** GLA variant classification according to the last updated (May 2025) version of the ACMG criteria and Clinvar databases. GLA variants classified as Pathogenic/Likely Pathogenic/Conflicting were considered to calculate the prevalence of AFD diagnosis. Variants reported with DNA nomenclature are not coding.
**Table S2:** Studies excluded at final screening.
**Table S3:** Risk of bias of the included studies according to the quality assessment tool appropriate for prevalence studies proposed by Hoy et al. [11].

## Data Availability

The data that support the findings of this study are available from the corresponding author upon reasonable request.
