# Supplementary material for: A Meta‐Analysis to Unveil the Diagnostic Gaps in Anderson–Fabry Disease in Women
Source: J Inherit Metab Dis. 2026 Feb 5;49(2):e70153. doi: 10.1002/jimd.70153 (PMC12876557; doi:10.1002/jimd.70153)

**Supplementary data**

**A Meta-analysis to Unveil the Diagnostic Gaps in Anderson-Fabry Disease in Women**

**L. Lenzini^1^, G. Pintus^2^, G. Gugelmo^3^, A.P. Burlina^4^, G.P. Fadini^3^, A.B. Burlina^5^ and N. Vitturi^3^**

**1 Department of Medicine, University of Padova, Italy**

**2 UOSD nefrologia e dialisi, Dip scienze mediche, Policlinico Tor Vergata, Roma, Italy**

3 Division of Metabolic Diseases, Department of Medicine, Padova University Hospital, University of Padova, Italy

4 Neurology Unit, St. Bassiano Hospital, Bassano del Grappa, Italy.

5 Division of Inherited Metabolic Diseases, Department of Women's

**Supplemental Table 1**. GLA variant classification according to the last updated (May 2025) version of the ACMG criteria and Clinvar databases. GLA variants classified as Pathogenic/Likely Pathogenic/Conflicting were considered to calculate the prevalence of AFD diagnosis. Variants reported with DNA nomenclature are not coding.

| **Study name** | **Pathogenic/Likely Pathogenic/Conflicting GLA Variant** | **Benign/of Uncertain Significance GLA Variant** |
| --- | --- | --- |
| **Afanasiev, 2020** | F113I |  |
| **Baptista, 2010** | R118C, D313Y | g.1136C>T |
| **Batta, 2017** | R363H |  |
| **Braga Silva, 2024** | I319T, A143T, R118C | ﻿T430G |
| **Brouns, 2010** | D313Y, A143T, S126G |  |
| **Elliott, 2011** | R118C, D244N, A143T, T410A |  |
| **Erdogmus, 2020** | A173T, D313Y |  |
| **Fancellu, 2015** | D313Y |  |
| **Fujii, 2009** | E66Q |  |
| **Gaspar, 2010** | D313Y, R118C | ﻿Val199GlyfsX203 |
| **Havndrup, 2010** | G271S | N139S |
| **Lanthier, 2017** | R118C, D313Y |  |
| **Leung, 2024** | M290T |  |
| **Lin, 2024** | T152H |  |
| **Malavera, 2020** | R118C |  |
| **Mallett, 2022** | G398Glyfs*5 |  |
| **Maron, 2018** | N215S |  |
| **Marquardt, 2012** | D313Y |  |
| **Merta 2006** | Q280K |  |
| **Moiseev, 2019** | R220X |  |
| **Monserrat, 2007** | A143T | L89P |
| **Nagamatsu, 2017** | M1T |  |
| **Nishino, 2012** | A73E , E66Q |  |
| **Ozpelit, 2023** | S126G |  |
| **Poli, 2017** | D313Y |  |
| **Reisin, 2018** | M296I, D313Y, ﻿S126G | c.-10C>T, c.-12G>A |
| **Reynolds, 2020** | L300F |  |
| **Romani R, 2024** | 801 + 1G, A143T, S126G,D313Y |  |
| **Romani, 2015** | L415P, Gly183lafs17* |  |
| **Saito, 2016** | E66Q |  |
| **Savostyanov, 2022** | T430Pheext*, A143T, D299E | ﻿N139S, L324W, c.897C |
| **Sens, 2024** | D313Y |  |
| **Terryn, 2008** | A143T |  |
| **Terryn, 2013** | A143T | ﻿c.639+6A>C |
| **Tomek, 2021** | D313Y, A143T, T412Sfs, R118C | ﻿﻿R38G, ﻿R30K |
| **Yalin, 2019** | D313Y, S126G, E59V, P205S | c.IVS6-10G>A, L8Q |
| **Yilmaz, 2017** | D313Y, S126G | ﻿ |
| **Zemanek, 2022** | N215S, L294* |  |

**Supplemental Table 2**. Studies excluded at final screening

| **Author,Year** | **Year** | **Referral Cause** | **Country** | **Continent** | **Screening Test** | **Reason for exclusion** |
| --- | --- | --- | --- | --- | --- | --- |
| Kalkan Uçar, 2012 | 2012 | CARDIAC | Turkey | EU | Enzymatic test | No female tested |
| Sachdev, 2002 | 2022 | CARDIAC | UK | EU | Enzymatic test | No female tested |
| Chimenti, 2004 | 2004 | CARDIAC | Italy | EU | Enzymatic test | No female tested |
| Mawatari, 2012 | 2012 | CARDIAC | Japan | ASIA | Enzymatic test | No female tested |
| Palecek, 2013 | 2013 | CARDIAC | Czech Republic | EU | Enzymatic test | No female tested |
| Kubo, 2017 | 2017 | CARDIAC | Japan | ASIA | Enzymatic test | No female tested |
| Kim, 2019 | 2019 | CARDIAC | Korea | ASIA | Enzymatic test | No female tested |
| Nakao, 1995 | 1995 | CARDIAC | Japan | ASIA | Enzymatic test | No female tested |
| Linthorst, 2003 | 2003 | RENAL | Netherland | EU | Enzymatic test | No female tested |
| Nakao, 2003 | 2003 | RENAL | Japan | ASIA | Enzymatic test | No female tested |
| Kleinert, 2009 | 2009 | RENAL | Austria | EU | Enzymatic test | No female tested |
| Doi, 2012 | 2012 | RENAL | Japan | ASIA | Enzymatic test | No female tested |
| Kalkan Uçar, 2012 | 2012 | RENAL | Turkey | EU | Enzymatic test | No female tested |
| Maruyama, 2013 | 2013 | RENAL | Japan | ASIA | Enzymatic test | No female tested |
| Veloso, 2018 | 2018 | RENAL | Brazil | AMERICA | Enzymatic test | No female tested |
| Vigneau, 2021 | 2021 | RENAL | France | EU | Enzymatic test | No female tested |
| Nagata, 2022 | 2022 | RENAL | Japan | ASIA | Enzymatic test | No female tested |
| Shimizu, 2022 | 2022 | RENAL | Japan | ASIA | Enzymatic test | No female tested |
| Wozniak, 2010 | 2010 | STROKE | USA | AMERICA | Enzymatic test | No female tested |
| Lin, 2018 | 2018 | RENAL | Taiwan | ASIA | Enzymatic test | No female tested |
| Martin, 2022 | 2022 | RENAL | Argentina | AMERICA | Enzymatic test | No female tested |
| Citro, 2022 | 2022 | CARDIAC | Italy | EU | Genetic test | VUS/B |
| Morita, 2006 | 2006 | CARDIAC | USA | AMERICA | Genetic test | VUS/B |
| Bersano, 2015 | 2015 | STROKE | Italy | EU | Genetic test | VUS/B |
| Fan, 2020 | 2020 | RENAL | China | ASIA | Enzymatic testATIC | VUS/B |
| Guzel,2023 | 2023 | CARDIAC | Turkey | EU | Genetic test | No description of GLA variant |
| Marrone, 2023 | 2023 | RENAL | Italy | EU | Genetic test | No description of GLA variant |
| Gwak, 2024 | 2024 | CARDIAC | Korea | ASIA | Genetic test | No description of GLA variant |

**Supplemental Table 3.** Risk of bias of the included studies according to the quality assessment tool appropriate for prevalence studies proposed by Hoy et al[11]

Definitions: Item 1: Was the study’s target population a close representation of the national population in relation to relevant variables, e.g. age, sex, occupation? Item 2: Was the sampling frame a true or close representation of the target population? Item 3: Was some form of random selection used to select the sample OR was a census undertaken? Item 4: Was the likelihood of non-response bias minimal? Item 5: Were data collected directly from the subjects (as opposed to a proxy)? Item 6: Was an acceptable case definition used in the study? Item 7: Was the study instrument that measured the parameter of interest (e.g. prevalence of low back pain) shown to have reliability and validity (if necessary)? Item 8: Was the same mode of data collection used for all subjects? Item 9: Was the length of the shortest prevalence period for the parameter of interest appropriate? Item 10: Were the numerator(s) and denominator(s) for the parameter of interest appropriate? Risk of bias: the overall risk of bias according to the ten items was defined according to the following definitions:

Low risk of bias: Further research is very unlikely to change our confidence in the estimate.

Moderate risk of bias: Further research is likely to have an important impact on our confidence in the estimate and may change the estimate.

High risk of bias: Further research is very likely to have an important impact on our confidence in the estimate and is likely to change the estimate.

| **Study name** | **Item 1** | **Item 2** | **Item 3** | **Item 4** | **Item 5** | **Item 6** | **Item 7** | **Item 8** | **Item 9** | **Item10** | **Risk of bias** |
| --- | --- | --- | --- | --- | --- | --- | --- | --- | --- | --- | --- |
| **Afanasiev, 2020** | No | Yes | No | NA | Yes | Yes | Yes | Yes | NA | Yes | Moderate |
| **Aladağ, 2023** | No | Yes | Yes | NA | Yes | Yes | Yes | Yes | NA | Yes | Moderate |
| **Arad, 2005** | No | Yes | Yes | NA | Yes | Yes | Yes | Yes | NA | Yes | Moderate |
| **Baptista, 2010** | Yes | Yes | Yes | NA | Yes | Yes | Yes | Yes | NA | Yes | Low |
| **Barman, 2019** | No | Yes | Yes | NA | Yes | Yes | Yes | Yes | NA | Yes | Moderate |
| **Batta, 2024** | Yes | No | Yes | NA | Yes | Yes | Yes | Yes | NA | Yes | Moderate |
| **Bekri 2005** | No | Yes | No | NA | Yes | Yes | Yes | Yes | NA | Yes | Moderate |
| **Braga Silva, 2024** | Yes | Yes | Yes | NA | Yes | Yes | Yes | Yes | NA | Yes | Moderate |
| **Brouns, 2007** | No | Yes | Yes | NA | Yes | Yes | Yes | Yes | NA | Yes | Moderate |
| **Brouns, 2010** | Yes | Yes | Yes | NA | Yes | Yes | Yes | Yes | NA | Yes | Low |
| **De Schoenmakere, 2008** | Yes | Yes | Yes | NA | Yes | Yes | Yes | Yes | NA | Yes | Low |
| **Dubuc, 2013** | No | Yes | Yes | NA | Yes | Yes | Yes | Yes | NA | Yes | Moderate |
| **Elliott, 2011** | Yes | Yes | Yes | NA | Yes | Yes | Yes | Yes | NA | Yes | Low |
| **Erdogmus, 2020** | Yes | Yes | Yes | NA | Yes | Yes | Yes | Yes | NA | Yes | Low |
| **Fancellu, 2015** | No | Yes | Yes | NA | Yes | Yes | Yes | Yes | NA | Yes | Moderate |
| **Fujii, 2009** | Yes | Yes | Yes | NA | Yes | Yes | Yes | Yes | NA | Yes | Low |
| **Gaspar, 2010** | Yes | Yes | Yes | NA | Yes | Yes | Yes | Yes | NA | Yes | Low |
| **Gündoğdu, 2017** | No | Yes | Yes | NA | Yes | Yes | Yes | Yes | NA | Yes | Moderate |
| **Hagège, 2011** | Yes | Yes | No | NA | Yes | Yes | Yes | Yes | NA | Yes | Moderate |
| **Härtl, 2022** | Yes | Yes | Yes | NA | Yes | Yes | Yes | Yes | NA | Yes | Low |
| **Havndrup, 2010** | No | Yes | Yes | NA | Yes | Yes | Yes | Yes | NA | Yes | Moderate |
| **Imasawa, 2023** | Yes | Yes | Yes | NA | Yes | Yes | Yes | Yes | NA | Yes | Low |
| **Jahan, 2020** | Yes | Yes | Yes | NA | Yes | Yes | Yes | Yes | NA | Yes | Low |
| **Kilarski, 2015** | Yes | Yes | Yes | NA | Yes | Yes | Yes | Yes | NA | Yes | Low |
| **Kinoshita N, 2018** | Yes | Yes | Yes | NA | Yes | Yes | Yes | Yes | NA | Yes | Low |
| **Kljajic, 2024** | Yes | Yes | Yes | NA | Yes | Yes | Yes | Yes | NA | Yes | Low |
| **Kotanko, 2004** | Yes | Yes | Yes | NA | Yes | Yes | Yes | Yes | NA | Yes | Low |
| **Lanthier, 2017** | Yes | Yes | Yes | NA | Yes | Yes | Yes | Yes | NA | Yes | Low |
| **Lau, 2025** | Yes | Yes | Yes | NA | Yes | Yes | Yes | Yes | NA | Yes | Low |
| **Lee, 2019** | Yes | Yes | Yes | NA | Yes | Yes | Yes | Yes | NA | Yes | Low |
| **Leung SP, 2024** | Yes | Yes | Yes | NA | Yes | Yes | Yes | Yes | NA | Yes | Low |
| **Lin Z, 2024** | Yes | Yes | Yes | NA | Yes | Yes | Yes | Yes | NA | Yes | Low |
| **Lin, 2025** | Yes | Yes | Yes | NA | Yes | Yes | Yes | Yes | NA | Yes | Low |
| [**Lv, 2009**](https://onlinelibrary.wiley.com/authored-by/Lv/YL) | Yes | Yes | Yes | NA | Yes | Yes | Yes | Yes | NA | Yes | Low |
| **Malavera, 2020** | No | Yes | No | NA | Yes | Yes | Yes | Yes | NA | Yes | Moderate |
| **Mallett, 2022** | Yes | Yes | Yes | NA | Yes | Yes | Yes | Yes | NA | Yes | Low |
| **Maron, 2018** | Yes | Yes | Yes | NA | Yes | Yes | Yes | Yes | NA | Yes | Low |
| **Marquardt, 2012** | Yes | Yes | Yes | NA | Yes | Yes | Yes | Yes | NA | Yes | Low |
| **Merta, 2006** | Yes | Yes | Yes | NA | Yes | Yes | Yes | Yes | NA | Yes | Low |
| **Moiseev, 2019** | Yes | Yes | Yes | NA | Yes | Yes | Yes | Yes | NA | Yes | Low |
| **Monserrat, 2007** | Yes | Yes | Yes | NA | Yes | Yes | Yes | Yes | NA | Yes | Low |
| **Nagamatsu, 2017** | Yes | Yes | Yes | NA | Yes | Yes | Yes | No | NA | Yes | Moderate |
| **Nishino T, 2012** | Yes | Yes | Yes | NA | Yes | Yes | Yes | Yes | NA | Yes | Low |
| **Okur, 2013** | Yes | Yes | Yes | NA | Yes | Yes | Yes | Yes | NA | Yes | Low |
| **Ozpelit, 2023** | Yes | Yes | Yes | NA | Yes | Yes | Yes | Yes | NA | Yes | Low |
| **Poli, 2017** | Yes | Yes | Yes | NA | Yes | Yes | Yes | Yes | NA | Yes | Low |
| **Reisin, 2018** | Yes | Yes | Yes | NA | Yes | Yes | Yes | Yes | NA | Yes | Low |
| **Reynolds, 2021** | Yes | Yes | Yes | NA | Yes | Yes | Yes | Yes | NA | Yes | Low |
| **Romani R, 2024** | Yes | Yes | Yes | NA | Yes | Yes | Yes | Yes | NA | Yes | Low |
| **Romani, 2015** | No | Yes | Yes | NA | Yes | Yes | Yes | Yes | NA | Yes | Moderate |
| **Sadasivan, 2020** | Yes | Yes | No | NA | Yes | Yes | Yes | No | NA | Yes | Moderate |
| **Saito O, 2016** | Yes | Yes | Yes | NA | Yes | Yes | Yes | Yes | NA | Yes | Low |
| **Sarikaya, 2012** | No | Yes | Yes | NA | Yes | Yes | Yes | Yes | NA | Yes | Moderate |
| **Savostyanov, 2022** | Yes | Yes | Yes | NA | Yes | Yes | Yes | Yes | NA | Yes | Low |
| **Sens, 2024** | Yes | Yes | Yes | NA | Yes | Yes | Yes | Yes | NA | Yes | Low |
| **Song, 2017** | Yes | Yes | Yes | NA | Yes | Yes | Yes | Yes | NA | Yes | Low |
| **Terryn, 2008** | Yes | Yes | Yes | NA | Yes | Yes | Yes | Yes | NA | Yes | Low |
| **Terryn, 2013** | Yes | No | No | NA | Yes | Yes | Yes | Yes | NA | Yes | Moderate |
| **Tomek, 2021** | Yes | Yes | Yes | NA | Yes | Yes | Yes | Yes | NA | Yes | Low |
| **Tran Vu MT, 2019** | No | Yes | Yes | NA | Yes | Yes | Yes | Yes | NA | Yes | Moderate |
| **Turkmen, 2016** | Yes | Yes | Yes | NA | Yes | Yes | Yes | Yes | NA | Yes | Low |
| **Yalin, 2019** | Yes | Yes | Yes | NA | Yes | Yes | Yes | Yes | NA | Yes | Low |
| **Yamashita S, 2019** | Yes | Yes | Yes | NA | Yes | Yes | Yes | Yes | NA | Yes | Low |
| **Yeniçerioğlu 2017** | Yes | Yes | Yes | NA | Yes | Yes | Yes | Yes | NA | Yes | Low |
| **Yilmaz, 2017** | Yes | Yes | Yes | NA | Yes | Yes | Yes | Yes | NA | Yes | Low |
| **Zemanek, 2022** | Yes | Yes | Yes | NA | Yes | Yes | Yes | Yes | NA | Yes | Low |
| **Zizzo, 2018** | Yes | Yes | Yes | NA | Yes | Yes | Yes | Yes | NA | Yes | Low |

Supplementary Figure 1. Funnel plot analysis of all (panel A) studies and of studies classified according to the enzymatic (panel B) and genetic (panel C) screening protocol. All meta-analytical calculations were performed using Comprehensive Meta-analysis 4 software.


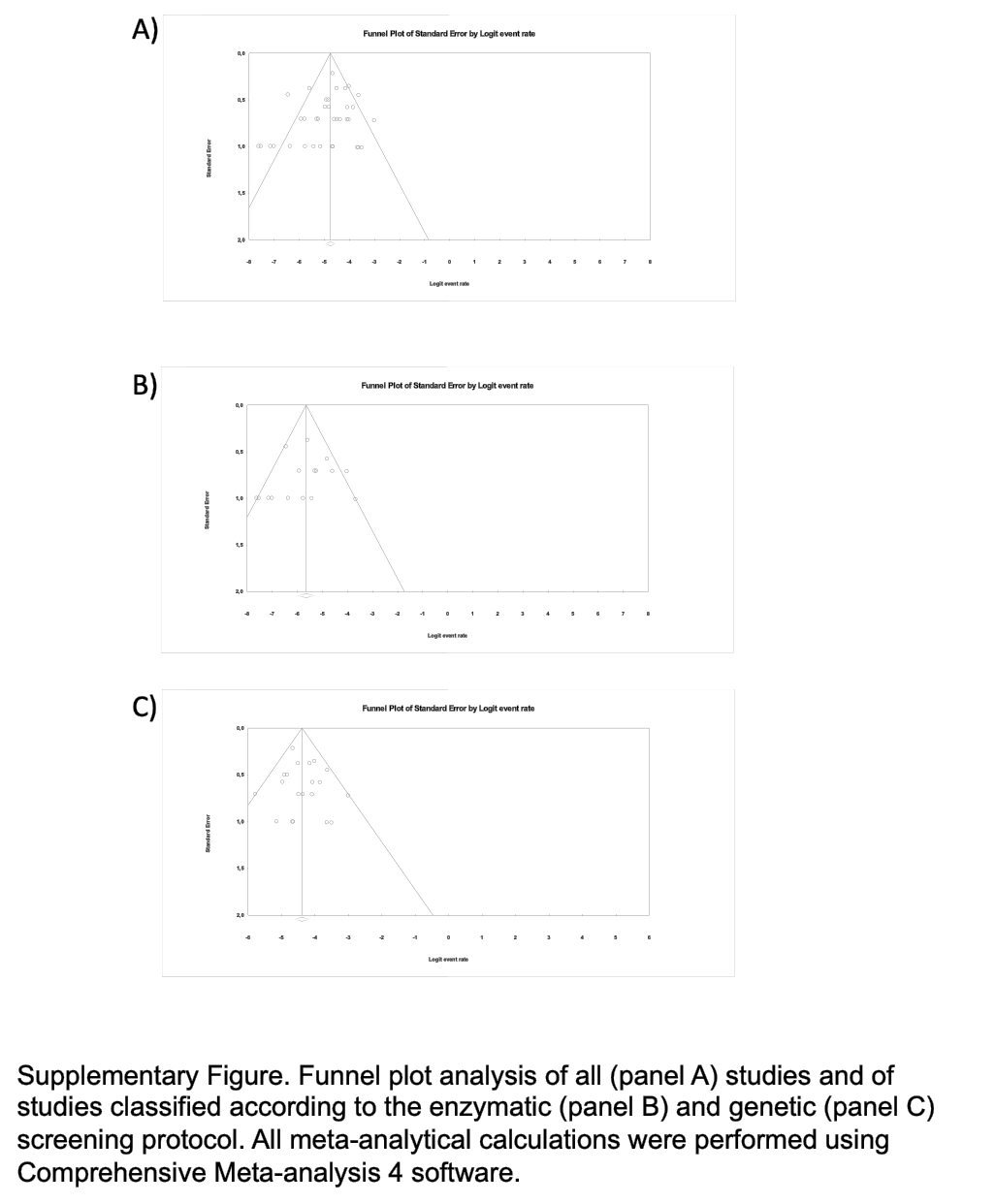

Supplement: Supplementary file 1 — Figure S1: Funnel plot analysis of all (A) studies and of studies classified according to the enzymatic (B) and genetic (C) screening protocol. All meta‐analytical calculations were performed using Comprehensive Meta‐analysis 4 software. Table S1: GLA variant classification according to the last updated (May 2025) version of the ACMG criteria and Clinvar databases. GLA variants classified as Pathogenic/Likely Pathogenic/Conflicting were considered to calculate the prevalence of AFD diagnosis. Variants reported with DNA nomenclature are not coding. Table S2: Studies excluded at final screening. Table S3: Risk of bias of the included studies according to the quality assessment tool appropriate for prevalence studies proposed by Hoy et al. [11]. [file JIMD-49-0-s001.docx]
